# Cell Kinetic Studies Fail to Identify Sequentially Proliferating Progenitors as the Major Source of Epithelial Renewal in the Adult Murine Prostate

**DOI:** 10.1371/journal.pone.0128489

**Published:** 2015-05-29

**Authors:** Jean-Christophe Pignon, Chiara Grisanzio, Ingrid Carvo, Lillian Werner, Meredith Regan, E. Lynette Wilson, Sabina Signoretti

**Affiliations:** 1 Department of Pathology, Brigham and Women’s Hospital, Boston, Massachusetts, United States of America; 2 Harvard Medical School, Boston, Massachusetts, United States of America; 3 Department of Medical Oncology, Dana-Farber Cancer Institute, Boston, Massachusetts, United States of America; 4 Biostatistics and Computational Biology, Dana-Farber Cancer Institute, Boston, Massachusetts, United States of America; 5 Departments of Cell Biology and Urology, School of Medicine, New York University, New York, New York, United States of America; Thomas Jefferson University, UNITED STATES

## Abstract

There is evidence that stem cells and their progeny play a role in the development of the prostate. Although stem cells are also considered to give rise to differentiated progeny in the adult prostate epithelium ex vivo, the cohort of adult prostate stem cells in vivo as well as the mechanisms by which the adult prostate epithelium is maintained and regenerated remain highly controversial. We have attempted to resolve this conundrum by performing in vivo tracing of serially replicating cells after the sequential administration of two thymidine analogues to mice. Our results show that, during normal prostate homeostasis, sequentially proliferating cells are detected at a rate that is consistent with a stochastic process. These findings indicate that in vivo, under steady-state conditions, most adult prostate epithelial cells do not represent the progeny of a small number of specialized progenitors that generate sequentially replicating transit-amplifying (TA) cells but are formed by stochastic cell division. Similarly, no rapidly cycling TA cells were detected during regeneration following one cycle of androgen-mediated involution/regeneration of the prostate epithelium. These findings greatly enhance our understanding of the mechanisms regulating prostate epithelial cell renewal and may have significant implications in defining the cell of origin of proliferative prostatic diseases.

## Introduction

It is widely accepted that cancer arises through a series of mutations that occur over a prolonged time period. Since adult stem/progenitor cells are long-lived cells, with a high proliferative capacity, they are able to accumulate multiple mutations and are considered to be the target cells for neoplastic transformation. However, this model is challenged by the evidence that, in contrast to rapidly proliferating epithelial cell compartments (e.g., epidermis and intestinal epithelium), slowly proliferating adult tissues (e.g., pancreatic epithelium and cardiac myocytes) can be maintained by random duplication of differentiated cells, with no significant contribution from stem/progenitor cells [1–3]. This observation suggests the possibility that such differentiated cells, which have the ability to self-duplicate, might also serve as target cells for carcinogenesis.

The adult prostate epithelium consists of luminal and basal cells residing on a basement membrane adjacent to smooth muscle cells and fibroblasts. Rare neuroendocrine cells are also present in the epithelium. By performing genetic lineage tracing studies of the prostate epithelium during both pre- and post-natal development, our group and others have convincingly shown that cells expressing a basal phenotype represent stem cells that are able to give rise to the different cell lineages of the prostate epithelium [4, 5]. However, the mechanisms regulating the maintenance and regeneration of the adult prostate epithelium remain unclear. Recent lineage tracing studies in adult mice suggest that basal progenitor cells do not play a significant role in normal prostate homeostasis or androgen-mediated regeneration of the prostate epithelium [6, 7]. While these novel findings suggest that the basal and luminal cell lineages become self-sustaining during adult life, it remains to be clarified whether each cell compartment (i.e. basal and luminal) is supported by a small pool of specialized progenitors that generate serially replicating transit amplifying (TA) cells, or by random duplication of adult epithelial cells.

**Fig 1:**
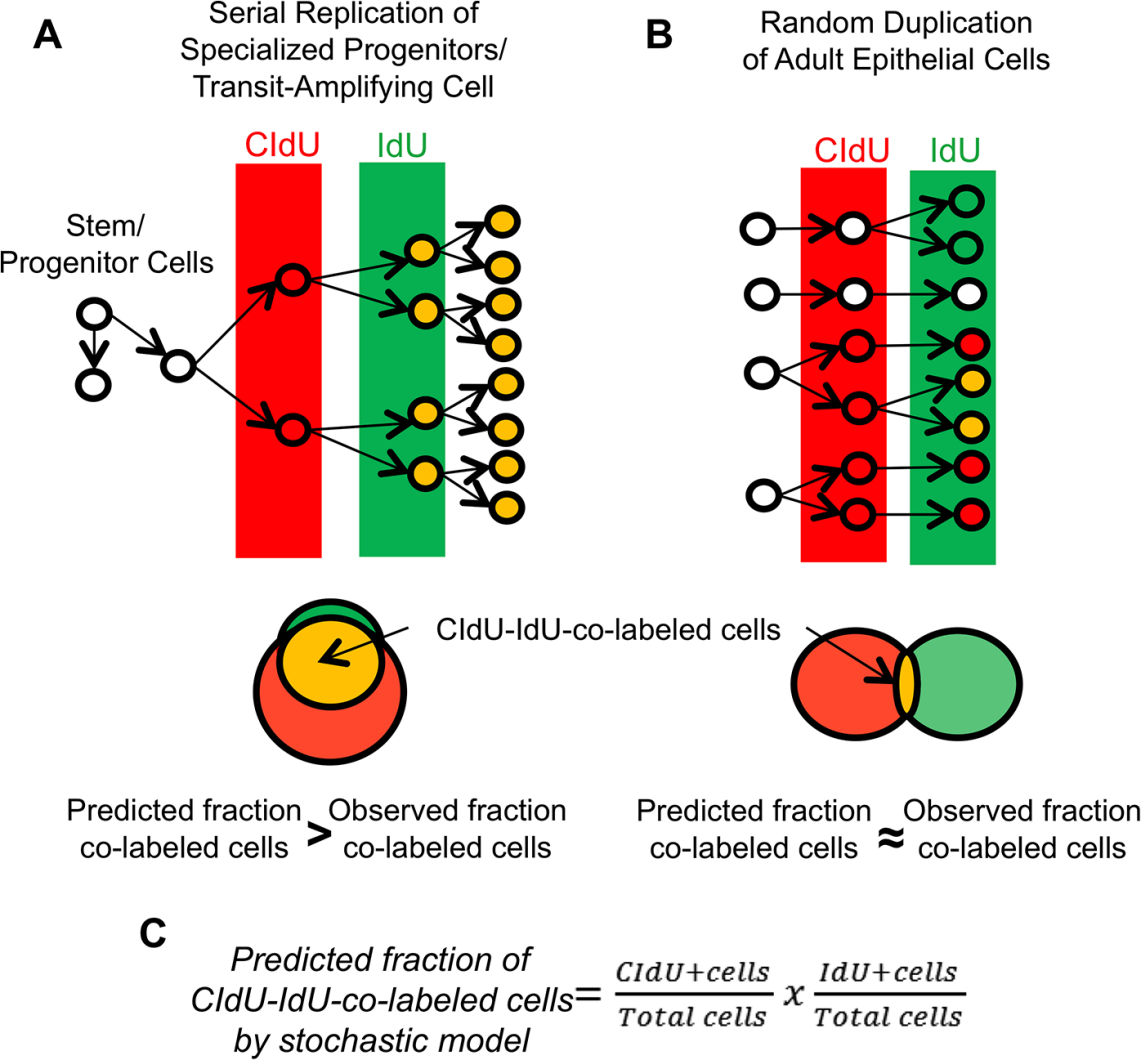
Experimental design. Consecutive administration of two different thymidine analogs, namely CIdU (in red) and IdU (in green), to mice allows the detection of serially replicating cells (CIdU-IdU-co-labeled cells, in orange). **(A):** According to the experimental methodology developed by Teta et al. [3], if the prostate epithelium is maintained by transit-amplifying (TA) cells that undergo consecutive cycles of cell division, a significant number of proliferating cells would be co-labeled by both thymidine analogs. In that case, the observed fraction of co-labeled cells should be greater than predicted fraction of co-labeled cells by the stochastic model. **(B):** In contrast, if the epithelium is maintained by cell division that occurs stochastically, the majority of replicating cells should be labeled with either one or the other thymidine analog. In this situation, the observed fraction of co-labeled cells should similar to the fraction of co-labeled cells predicted by the stochastic model. **(C):** The fraction of co-labeled cells predicted by the stochastic model is calculated by multiplying the fraction of CIdU-labeled cells by the fraction of IdU-labeled cells. This figure has been adapted from Humphreys et al. [8].

In order to discriminate between these two possibilities, we employed an unbiased DNA-analog based approach successfully used in different organs (i.e. pancreas, kidney, brain, heart) to track multiple rounds of cell division *in vivo*. Experiments are based on the sequential administration of two different thymidine analogs, 5-chloro-2-deoxyuridine (CldU) and 5-iodo-2-deoxyuridine (IdU) to adult mice [3, 8]. If the prostate epithelium is maintained by a small pool of specialized progenitors that give rise to TA cells, the majority of cells labeled with the second analog (IdU) would have previously proliferated and thus be co-labeled with CIdU (Fig 1A). On the contrary, if the prostate epithelium is sustained by random epithelial cell division, the majority of replicating cells would be single labeled with either CIdU or IdU (Fig 1B). In this scenario, the fraction of CIdU-IdU-co-positive cells would be relatively small and would mirror the fraction predicted by the stochastic model (Fig 1C).

## Materials and Methods

### Materials and mice

CldU (Sigma) and IdU (MP Biomedicals) were dissolved at 1mg/ml [3] and stored at 4°C for no longer than two weeks. Thymidine analog solutions were administrated via drinking water and were protected from light. For labeling periods longer than 1 day, thymidine analog solutions were renewed every other day. Experimental mice were C57BL6/J males (Jackson Laboratories), 7-week-old at the beginning of administration of thymidine analogs.

### Castration/Regeneration experiments

Five-week-old male mice were castrated by gonadectomy to cause prostate regression. Two weeks later, prostate regeneration was induced by subcutaneous implantation of a testosterone pellet (12.5 mg released over 60 days at a rate of ~0.21 mg/day, Innovative Research of America) [9]. This study was carried out in strict accordance with the recommendations in the Guide for the Care and Use of Laboratory Animals of the National Institutes of Health. The protocol was approved by the Harvard Medical School Center for Animal Resources and Comparative Medicine Standing Committee (protocol # 04066). All surgeries were performed under Isofluran anesthesia, and all efforts were made to minimize suffering.

### Tissue preparation

Mice were sacrificed immediately at the end of the labeling period of the second thymidine analog (IdU). Mice were perfused by intra-cardiac injection of phosphate buffered saline (PBS), followed by 10% formalin. The prostate lobes and the prostatic urethra were dissected en bloc using a dissecting microscope, fixed in 10% formalin for 48 hours, dehydrated and then embedded in paraffin so that the long axis of the anterior prostate lobes were parallel to the surface of the paraffin block (i.e. the plane of section). Serial sections were performed throughout the blocks. Proximal regions of prostatic ducts were identified in H&E stained tissue sections on the basis of their proximity to the urethra, evidence of multiple smooth muscle cell layers and presence of luminal cells with low-columnar or cuboidal morphology [10–12]. Distal/intermediate regions of prostatic ducts were identified on the basis of their distal/intermediate position along the ducts in relation to the urethra, and by the presence of tall-columnar luminal cells. Immunofluorescence staining was performed on tissue sections adjacent to the H&E stained sections to ensure accurate differentiation of the distal/intermediate from the proximal regions of ducts.

### Immunofluorescence studies

Four micron-thick tissue sections were deparaffinized and rehydrated in water, and antigen retrieval was done by heating slides in citrate buffer (pH 6.0) in a pressure cooker at 125°C for 30 seconds, followed by 20 minutes of cooling. Sections were sequentially blocked for 10 minutes with a serum-free blocking reagent (Biocare) then incubated with rabbit polyclonal anti-keratin 14 (Krt14) antibody (1:80,000, AF64, Biolegend) for 1 hour at room temperature. The C-terminus of the mouse Krt14 protein was used as antigen to raise this antibody [13]. Sections were then washed for 5 minutes in PBS, and incubated with species-specific HRP-conjugated secondary antibody for 1 hour. Sections were then incubated with Cy5 TSA amplification kit (1:50, Perkin Elmer) for 5 minutes. Following a wash in PBS for 5 minutes, sections were incubated overnight at 4°C with a mouse monoclonal anti-BrdU antibody shown to have a high affinity for IdU (1:100, clone B44, BD Biosciences). Iodouridine was used as antigen to raise this antibody [14]. Sections were then washed for 5 minutes in PBS, incubated for 20 minutes at 37°C on a 220 rpm shaking platform with pre-heated (at 37°C) high salt Tris Buffer Saline and Tween20 solution (TBST, 0.5 M NaCL, 36mM Tris-HCl, 0.5% Tween 20, pH 8), then rinsed again for 10 minutes in PBS. Subsequently sections were incubated with a rat monoclonal anti-BrdU antibody shown to have a high affinity for CIdU for 1 hour at room temperature (1:300, clone BU1/75, Accurate Chemical & Scientific Corporation). The information regarding the antigen used to raise this antibody is undisclosed. Sections were washed for 5 minutes in PBS, incubated for 20 minutes at 37°C on a 220-rpm shaking platform with pre-heated (at 37°C) high salt TBST buffer, then rinsed again for 10 minutes in PBS. Finally, tissue sections were incubated with the secondary cocktail of goat antibodies, Cy2-anti-mouse and Cy3-anti-rat (Jackson ImmunoResearch, #115-225-166 and #112-225-167 respectively), both at 1:150 dilution for 1 hour at room temperature, and coversliped with prolong gold antifade mounting medium containing DAPI (Invitrogen). The specificity of the two anti-BrdU antibodies against IdU and CIdU was verified by performing double CIdU/IdU immunostaining on tissue sections of mice treated for one day with CIdU or IdU alone (S1 Fig). The specificity of the anti-Krt14 antibody (AF64, Biolegend) was validated in a previous study [13] and we confirmed that our protocol produced selective staining in prostate basal cells.

### Microscopy and cell count

Microscopic examination was performed on a Leica SP8 X inverted confocal microscope. For each anterior, dorsal and ventral lobe, at least five 400X fields were randomly selected within the distal/intermediate and proximal regions of prostatic ducts of 3 different mice, leading to a total of at least 15 fields per each prostatic region (proximal and distal/intermediate) per mouse. The selected fields were photographed using a 40X oil immersion objective. For both the distal/intermediate and the proximal regions of ducts, a minimum of 2000 epithelial cell nuclei per mouse (including at least 200 nuclei of Krt14-positive basal cells) were counted manually for IdU and CIdU positivity on composite images obtained with Fiji software [15]. The elongated nuclei of stromal cells located under the Krt14-positive basal cells were excluded from the analysis. The percentage of labeled nuclei for the proximal or distal/intermediate regions of ducts of each mouse was obtained by first calculating the percentage of labeled cells for each of the three lobes and then calculating the average of the three values. The mean percentage and standard deviation of labeled nuclei were then calculated on the basis of the percentages obtained from 3 different mice. For each mouse, the predicted stochastic fraction of double-labeled cells was obtained by multiplying the fraction of CIdU-positive cells by the fraction of IdU-positive cells (Fig 1C). The mean predicted stochastic fraction and standard deviation of labeled nuclei were then calculated on the basis of rates obtained from 3 different mice.

### Statistical analysis

Each experiment included 3 mice. A one-sided paired t-test was used to assess whether the observed fraction of double-labeled cells was higher than the fraction of double-labeled cell predicted by a stochastic model. A nominal p-value (p) <0.05 was considered as statistically significant; there was no adjustment for multiple comparisons.

## Results

### The physiologic turn-over of the adult prostate epithelium depends on random epithelial cell division

**Fig 2:**
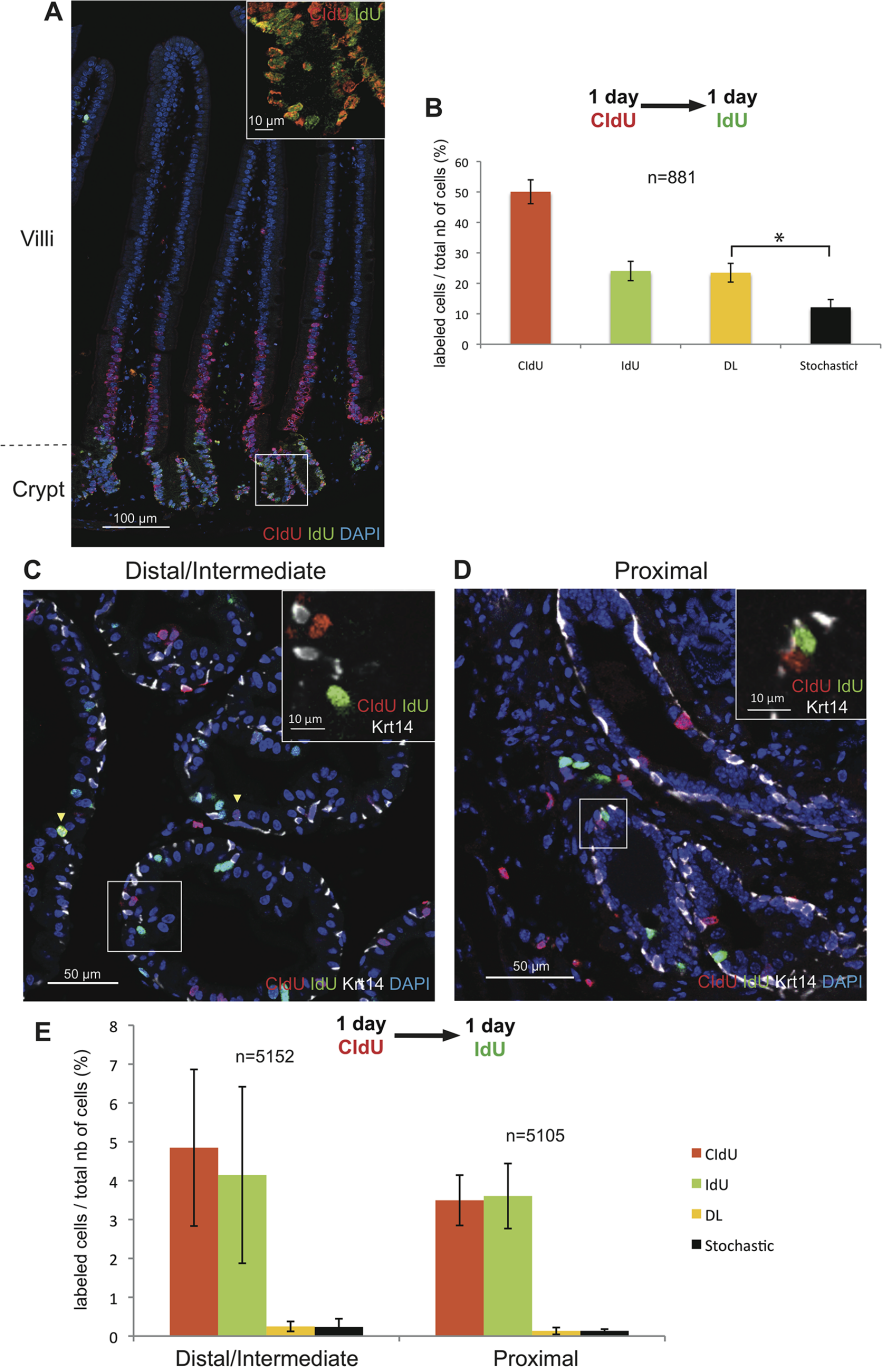
Renewal of the adult prostate epithelium does not depend on rapidly serially proliferating progenitor/TA cells. Detection of rapidly proliferating progenitors was performed on 7-week-old mice sequentially treated with CIdU and IdU for 1 day each. Mice were sacrificed immediately after the end of IdU administration. **(A):** Representative images of double immunofluorescence staining for CIdU and IdU in a section of small intestine demonstrating the presence of double-labeled progenitor cells in the intestinal crypt (inset). **(B):** Graphic representation of the percentages of intestinal epithelial cells labeled with CIdU, IdU, or CIdU/IdU. The predicted stochastic fraction is also shown. Data represent the means *±* SD for three mice per group. **(C, D):** Representative images of triple immunofluorescence staining for CIdU, IdU and Krt14 performed on sections of the distal/intermediate region of ducts **(C)** and the proximal region of ducts **(D)** of the dorsal prostate showing that the majority of cells are single labeled (inset). Yellow arrowheads indicate CIdU-IdU-co-labeled cells. **(E):** Graphic representation of the percentages of epithelial prostate cells (both basal and luminal) labeled with CIdU, IdU, or CIdU/IdU (DL). The predicted stochastic fraction is also shown. Data represent the means *±* SD for three mice per group. n indicates the average number of nuclei counted per mouse.* indicates p<0.05.

We first demonstrated our ability to detect cells undergoing sequential cell divisions by analyzing the epithelium of the small intestine that is maintained by progenitor cells that give rise to rapidly proliferating TA cells located in the crypts [3, 16]. Adult mice were sequentially treated with CIdU and IdU (administered through drinking water) for one day each and sacrificed immediately after IdU administration. CIdU and IdU were detected by immunofluorescence under a validated protocol. Comparable incorporation rates of CIdU and IdU were also verified before proceeding with analysis (S1 Fig). Consistent with previously published data [3], many of the epithelial cells in the intestinal crypts were co-labeled (Fig 2A). IdU-labeled cells were mainly co-labeled with CldU and resided in the crypts, while cells positive for CldU alone were localized mainly above the double-labeled cells along the lower part of the villi. This indicated that the first labeling period marked both sequentially proliferating cells (double-labeled) and cells that exited the cell cycle to migrate up the villi (CldU-labeled). In line with these results, quantitative analysis demonstrated that the observed fraction of double-labeled cells was significantly higher than the fraction of double-labeled cells that would have been observed if the epithelium were sustained by cells replicating in a stochastic manner (23.5±3.2% versus 12.1±2.6%; p<0.05) (Fig 2B). Taken together, these data confirm that serial labeling of proliferating cells with two different thymidine analogs is a valid method to detect serially replicating TA cells.

We then evaluated the presence of rapidly proliferating TA cells in the prostate epithelium by studying the distal/intermediate and proximal regions of prostate ducts of 7-week-old mice treated consecutively with CIdU and IdU, for 1 day each. Prostate tissue sections were immunostained for the two thymidine analogs as well as for the basal cell marker Krt14 in order to differentiate basal cells from Krt14-negative luminal cells. Analysis of epithelial (both luminal and basal) cells of the distal/intermediate regions of the prostate ducts revealed that 4.8±3.5% were labeled with CldU and 4.1±3.6% were labeled with IdU. The fraction of double-labeled cells, was not significantly higher compared to the fraction of cells predicted by the stochastic model (0.2±0.1%) (p = 0.4) (Fig 2C and 2E). Similarly, the observed fraction of CIdU-IdU-co-labeled cells (0.1±0.1%) was not significantly higher than the predicted fraction (0.1±0.004%) in the proximal region of prostatic ducts (p = 0.5) (Fig 2D and 2E). Moreover, CIdU- and IdU-co-expressing cells were not enriched in either the basal or luminal cell compartment of either the distal/intermediate or the proximal regions of ducts (S2 Fig). It should be noted that in contrast to the intestinal epithelium, the fraction of prostate epithelial cells labeled with the first thymidine analogue (CldU) does not represent double of the fraction of cells labeled with the second analogue (IdU). This result suggests that most of the cells that have undergone DNA replication in day 1 of treatment have not completed mitosis at the end of day 2, implying that prostate epithelial cells have a longer cell cycle time compared to intestinal cells. As a consequence, the 1-day CldU/1-day IdU labeling period is likely inadequate for detecting progenitors/TA cells in the quiescent prostate epithelium and longer treatment periods that allow the labeling of cells that sequentially proliferate at a slower rate are required.

**Fig 3:**
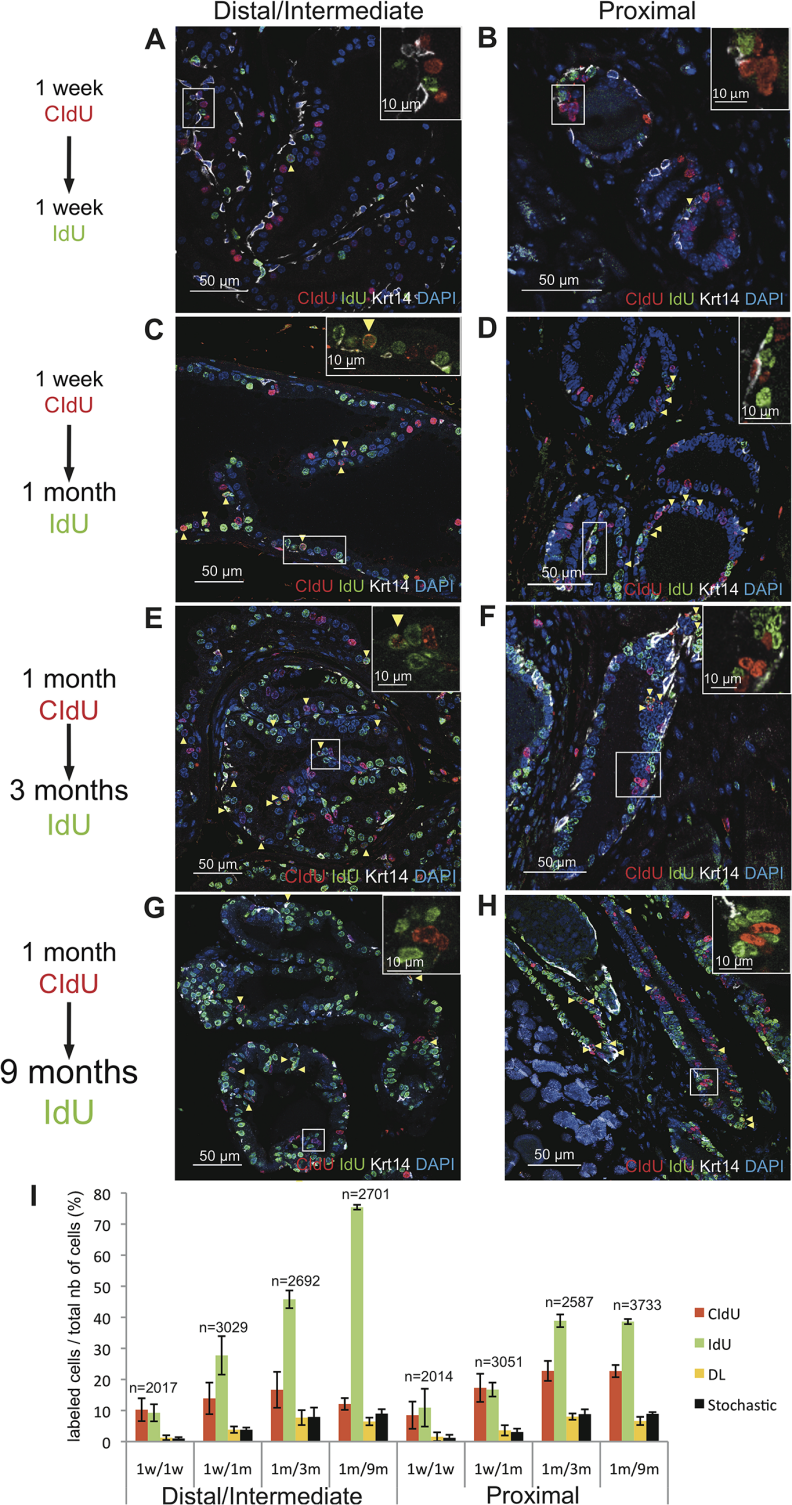
Renewal of the adult prostate epithelium does not depend on slowly serially proliferating progenitor/TA cells. Representative images of triple immunofluorescence staining for CIdU, IdU and Krt14 on sections of the distal/intermediate region **(A, C, E, G)** and the proximal region of ducts **(B, D, F, H)** of the dorsal prostate of 7-week-old mice treated for either 1 week with CIdU followed by 1 week of IdU **(A, B), or** 1 week with CIdU followed by 1 month of IdU **(C, D)**, or for 1 month of CIdU followed by 3 months of IdU **(E, F)**, or for 1 month of CIdU followed by 9 months of IdU **(G, H)**. In all experiments, mice were sacrificed immediately after the end of IdU administration. Yellow arrowheads indicate double-labeled cells while insets show that the majority of the cells are single labeled. **(I):** Graphic representation of the percentages of epithelial prostate cells (both basal and luminal) labeled with CIdU, IdU, or CIdU/IdU (DL) for the indicated treatment groups. The predicted stochastic fraction is also shown. Data represent the means *±* SD for three mice per group. n indicates the average number of nuclei counted per mouse.

To detect TA cells that have relatively longer cell cycle time and might remain quiescent for variable time periods before reentering the cell cycle, CldU was administered to 7-week-old mice for either 1 week or 1 month, followed by prolonged (1 week to 9 months) treatment with IdU. The different treatment groups were as follows: 1 week of CIdU labeling followed by 1 week of IdU labeling, 1 week of CIdU labeling followed by 1 month of IdU labeling, 1 month of CIdU labeling followed by 3 months of IdU labeling, and 1 month of CIdU labeling followed by 9 months of IdU labeling. Quantitative analysis was performed in both distal/intermediate and proximal regions of ducts as well in the in the basal and luminal cell compartments. In all the treatment groups, the fraction of CIdU-IdU-co-labeled cells was not significantly higher than the fraction predicted by the stochastic model (Fig 3). Specifically, the observed fraction of CIdU-IdU-co-labeled cells versus (vs) the predicted fraction of CIdU-IdU-co-labeled for the different labeling groups were: 1.3±0.8% vs 1.0±0.4% (p = 0.18) for the 1-week/1- week labeling group in the distal/intermediate regions of ducts, and 1.5±1.3% vs 1.0±0.9% (p = 0.11) for the 1-week/1-week labeling group in the proximal regions of ducts; 3.8±1.0% vs 3.8±0.7% (p = 0.4) for the 1-week/1-month labeling group in the distal/intermediate regions of ducts, and 3.6±1.6 vs 3.1±1.1% (p = 0.1) for the 1-week/1-month labeling group in the proximal regions of ducts; 7.7±2.4% vs 7.9±1.2% (p = 0.6) for the 1-month/3-month labeling group in the distal/intermediate ductal regions, and 8.0±1.1% vs 8.8±1.5% (p = 0.9) for the 1-month/3-month labeling group in the proximal regions of ducts; 6.5±1.2% vs 9.0±1.4% (p = 0.9) for the for the 1-month/9-month labeling group in the distal/intermediate regions of ducts, and 6.6±1.3% vs 8.9±0.5% (p = 0.9) for the 1-month/9-month labeling group in the proximal regions of ducts. Of note, no enrichment of co-labeled cells was observed in either the basal or luminal cell compartments of either distal/intermediate or proximal ductal regions (S3 Fig).

In order to confirm that sequential cell division occurs stochastically in the adult prostate epithelium, we utilized a complementary approach based on the treatment of mice with CldU for 2 weeks, followed by variable washout periods (5 days, 2 months, 4 months or 10 months) and subsequent treatment with IdU for another 2 weeks (S4 Fig). Similarly to what was observed in the previous experiment, evaluation of the epithelium of the distal/intermediate regions of ducts revealed that in all the treatment groups the number of co-labeled cells was very low compared to the CIdU or IdU labeled cells, and that the fraction of CIdU-IdU-co-positive cells was never higher when compared to the fraction predicted by the stochastic model (S4 Fig). Specifically, the observed fraction of CIdU-IdU-co-labeled cells versus the predicted fraction of CIdU-IdU-co-labeled for the groups were respectively: 5.9±0.8% vs 6.8±1.3% (p = 0.9) for the 5 days washout group, 4.0±1.7% vs 5.1±1.3% (p = 0.9) for the 2 months washout group, 1.3±0.5% vs 2.4±0.2% (p = 0.9) for the 4 months washout group, and 0.6±0.5% vs 0.9±0.4% (p = 0.9) for the 10 months washout group.

These data indicate that the renewal of prostate epithelial cells during adult life is not sustained by a small pool of specialized progenitors that give rise to serially replicating TA cells, (Fig 1A) but depends on random replication of both basal and luminal cells (Fig 1B).

### Androgen mediated regeneration of the prostate epithelium does not involve rapidly proliferating TA cells

**Fig 4:**
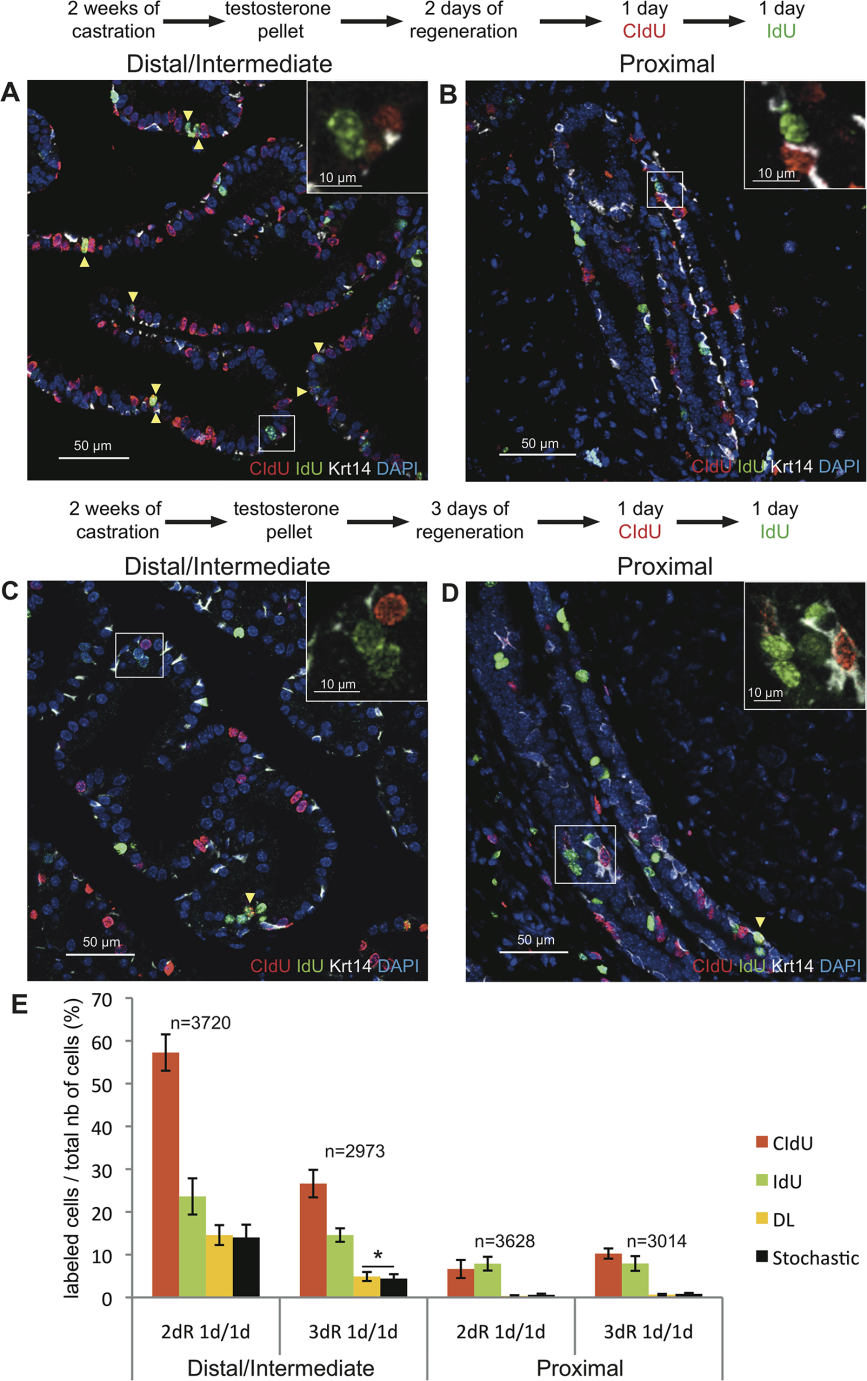
Androgen mediated regeneration of the prostate epithelium after castration does not depend on rapidly serially proliferating progenitor/TA cells. Representative images of triple immunofluorescence staining for CIdU, IdU and Krt14 on sections of the distal/intermediate **(A, C)** and the proximal **(B, D)** regions of ducts from the dorsal prostate of castrated mice sequentially treated with CIdU and IdU (1 day each) at day 2 **(A, B)** or day 3 **(C, D)** after androgen supplementation. In all experiments, mice were sacrificed immediately after the end of IdU administration. Yellow arrowheads indicate double-labeled cells while insets show that the majority of the cells are single labeled. **(E):** Graphic representation of the percentages of epithelial prostate cells (both basal and luminal) labeled with CIdU, IdU, or CIdU/IdU (DL) for the indicated treatment groups. 2dR and 3dR indicate mice that were treated with the thymidine analogs at day 2 or day 3 after androgen supplementation, respectively. The predicted stochastic fraction is also shown. Data represent the means ± SD for three mice per group. n indicates the average number of nuclei counted per mouse. * indicates p<0.05.

We utilized a well-characterized model of androgen-mediated involution/regeneration of the prostate [17, 18] to assess the contribution of sequentially replicating TA cells to the regeneration of the prostate epithelium after injury. Cell damage was induced in the prostate epithelium by castration, which is known to cause extensive apoptosis. Two weeks after castration, the prostate epithelium was regenerated by subcutaneous implantation of testosterone pellets. Mice were treated consecutively with CIdU and IdU for 1 day each, starting at either 2 days or 3 days after androgen supplementation, when prostate epithelial cells are known to be actively proliferating [19, 20]. In mice in which the treatment was started 2 days after pellet implantation, the percentage of cells in the distal/intermediate regions of ducts labeled with the thymidine analogs was high, reaching 57.2±3.2% for CIdU- and 23.0±1.6% for IdU-labeled cells (Fig 4A and 4E). Although these percentages were very close to the percentages of labeled cells that we observed in the intestine in the 1 day-1 day experiment (Fig 2B), CIdU-IdU-co-labeled cells were observed considerably less frequently in the prostate (14.6±1.0%) compared to the intestine (23.5±3.2%). Moreover, the observed fraction of co-labeled cells was not significantly higher compared to the fraction predicted by the stochastic model (14.0±1.0%) (p = 0.2). Of note, the overall percentage of cells labeled with thymidine analogs was significantly lower in the proximal regions of ducts compared to the distal/intermediate ductal regions (p<0.05). However, similarly to what was observed in the distal/intermediate regions, the fraction of co-labeled cells was not higher than the fraction predicted by the stochastic model (p = 0.8) (Fig 4B and 4E). No enrichment of co-labeled cells was observed in either the basal or luminal cell compartments of either distal/intermediate or proximal ductal regions (S5 Fig).

In comparison to mice that started treatment with thymidine analogs 2 days after testosterone supplementation, mice treated 3 days after androgen administration showed a decrease in the fractions of CldU- and IdU-labeled cells in the distal/intermediate regions of ducts but not in the proximal ductal regions. We observed that the fraction of CIdU-IdU-co-labeled cells in distal/intermediate ductal regions (4.9±1%) was higher than the fraction predicted by the stochastic model (4.4±1%) (p<0.05) (Fig 4C and 4E). However, in contrast to what observed in the intestine the difference was very modest and only a minority of IdU-labeled cells was co-labeled with CldU. In the proximal ductal regions, the fraction of co-labeled cells (0.6±0.1%) was not higher than the predicted fraction (0.8±0.2%) (p = 0.7) (Fig 4D and 4E). Similar results were observed when the analyses were performed in the basal and luminal cell compartments separately (S5 Fig).

In summary, our studies fail to identify the existence of rapidly cycling TA cells in the prostate epithelium that regenerates in response to androgen replenishment.

## Discussion

In this manuscript, we demonstrate for the first time that random duplication of prostate epithelial cells rather than serial division of a small pool of progenitor/TA cells is the dominant mechanism of epithelial cell replacement during normal homeostasis of the prostate epithelium.

Previous cell kinetic studies of the adult prostate epithelium have shown that proliferating cells as well long-term label-retaining cells can be detected in both basal and luminal cell compartments [17, 19, 21, 22]. While these results raise the possibility that basal and luminal cell types might be independent of each other for renewal, recent cell-specific lineage tracing of basal and luminal cells performed in the mouse prostate have validated this hypothesis, convincingly demonstrating that basal cells do not contribute significantly to the pool of luminal cells and vice versa [6, 7, 23]. As a consequence, it has been postulated that each cell compartment is replenished by unipotent stem/progenitor cells that give raise to TA cells [4]. In contrast to this model, our analysis fails to identify sequentially proliferating TA cells as the major source of prostate epithelial cells that are formed during adult life. While our results are somewhat surprising, they are in line with recent data documenting that the turn-over of slowly proliferating adult organs, such as the pancreas and the heart muscle, depends on the stochastic proliferation of differentiated cells [1–3]. We recognize, however, that the mechanisms sustaining the renewal of the mouse and the human prostate epithelium in vivo might be different, and stem/progenitor cells might play a role in the human prostate. In line this possibility, two independent studies have shown that cytochrome c oxidase deficiency associated with specific mitochondrial DNA (mtDNA) mutations occurs focally in both basal and luminal cell compartments of the normal human prostate epithelium [24, 25]. Although it is unclear whether mtDNA mutations occur during adulthood, these data suggest that the renewal of adult human basal and luminal prostate cells depends on a common progenitor/stem cell.

It has been proposed that stem/progenitor cells undergo selective segregation of their template DNA strand during mitosis when the newly synthesized DNA strand segregates in the daughter cell but not in the stem cell [26]. Consecutive administration of thymidine analogs would therefore fail to efficiently label proliferating stem cells. It should be noted that this hypothesis is highly controversial [27], but even if asymmetric DNA strand segregation took place in the stem cell compartment of the prostate epithelium, this occurrence would not likely affect the results of our studies. Indeed, our experiments are aimed at labeling the committed TA cells (not the stem cells) that are not thought to be subject to asymmetrical DNA strand segregation [28, 29]. Moreover, the results we obtained in the small intestine demonstrate that TA cells can be identified using this methodology.

Several studies have demonstrated the existence of facultative stem cells, namely cells that acquire stem cell capacities only after stimulation. As an example, in the liver, oval cells are thought to represent facultative stem cells that participate in liver regeneration only after severe liver damage [30]. In keeping with this concept, the cell type(s) that maintain normal prostate epithelial homeostasis might be different from the cell type(s) that sustain prostate cell renewal after cell injury. Since the survival of prostate epithelial cells depends on androgens, we utilized a model of androgen-mediated involution/regeneration of the prostate epithelium to induce cell injury and subsequent cell repair. However, prostate regeneration following the administration of androgens to castrated mice also failed to demonstrate the existence of rapidly proliferating TA cells. A previous study identified rare (present at an incidence of 0.7%) castration-resistant NKX3.1-expressing (CARN) pluripotent stem cells that have been proposed to serve as facultative stem cells that sustain prostate regrowth in orchiectomized mice upon androgen administration [31]. However, our data do not support this hypothesis and clearly show that two days after androgen supplementation, and over the subsequent 2 day-period the majority (~70%) of epithelial cells of the distal/intermediate regions of ducts have undergone cell division. Overall, our findings demonstrate that androgen mediated regeneration of the prostate epithelium is dependent on the replication of a large pool of epithelial cells that survive androgen withdrawal rather than on the amplification and differentiation of rare castration resistant prostate epithelial stem/progenitor cells such as CARNs.

In contrast to our in vivo data, several ex vivo analyses have shown that both human and mouse prostate epithelial cells expressing basal cell markers display stem cell abilities [32–41]. Of note, studies from independent groups have demonstrated that the proximal regions of murine prostate ducts are enriched for stem cells [32, 37, 40, 42]. Moreover, cell kinetic studies have indicated that long-term label-retaining (LRC) cells after multiple cycles of castration/regeneration, considered to be stem cells, are located in the proximal region of the ducts [40]. In line with these findings, we observed that the epithelial cells of proximal ductal regions proliferate less in response to androgens as compared to cells from the distal/intermediate ductal regions. However, we did not find any evidence that TA cells in proximal ductal regions had a significant role in cell renewal in the conditions used in these investigations. It should be noted that our analysis was performed after only one cycle of castration/regeneration and it is possible that facultative prostate stem cells may only be called upon for epithelial regeneration after multiple cycles of androgen depletion/replenishment once the capacity for division of more differentiated progeny has been exhausted. Moreover, since there is evidence that epithelial cell proliferation (DNA synthesis) starts around 24 hours after androgen treatment of the castrated prostate [19, 20], our analysis, performed 48 to 72 hours after androgen administration, could have also failed to detect early-activated TA cells.

It is likewise possible that facultative prostate stem cells could be activated under other types of stressful conditions. For example, similarly to what is observed during normal homeostasis, genetic lineage tracing studies suggest that basal cells do not contribute significantly to the replenishment of the luminal cell compartment during androgen-mediated involution/regeneration of the prostate [6, 7]. In contrast, Kwon et al [43] recently described a model of bacterial prostatitis that stimulates the proliferation and differentiation of basal cells into luminal cells. Thus, under certain conditions, basal cells may give rise to luminal cells in vivo, whereas under other circumstances basal and luminal cells may self-replicate. Further studies are needed to clarify the role of basal cells as facultative prostate stem cells and their possible role in tumorigenesis.

## Supporting Information

S1 Fig (A, B) The immunofluorescence protocol was optimized to ensure that the rat anti-BrdU and the mouse anti-BrdU antibodies selectively recognized CIdU and IdU, respectively. To this end, 7 week old male mice were treated for 1 day with CldU (A) or IdU (B) and small intestine tissue sections were double immunostained with the rat anti-BrdU antibody (high affinity for CIdU) and the mouse anti-BrdU antibody (high affinity for IdU) as described in the materials and methods section. The mouse anti-BrdU antibody produces no signal in mice treated with CldU (A, middle panel) and the rat anti-BrdU antibody produces no signal in mice treated with IdU (B, left panel). **(C):** Incorporation of the thymidine analogs, CldU and IdU occurs at a similar rate in the mouse prostate. Graphic representation of the percentages of epithelial prostate cells (both basal and luminal) labeled with either CIdU or IdU in 7 week old mice treated for 1 day with CldU or IdU. Data represent the mean *±* SD for three mice per group.
(TIF)

S2 Fig Rapidly proliferating progenitor/TA cells are not enriched in basal cell or luminal cell compartments of the prostate. Prostate tissue sections of 7 week old mice sequentially treated with CIdU and IdU for 1 day each were triple stained for CIdU, IdU and Krt14 and quantification of the labeled cells was performed in the Krt14-positive (basal) and the Krt14-negative (luminal) epithelial cell compartments. Here we show the graphic representation of the percentages of prostate cells labeled with CIdU, IdU, or CIdU/IdU in the basal or the luminal compartments of the distal/intermediate and proximal regions of prostatic ducts. The predicted stochastic fraction is also shown. Data represent the means *±* SD for three mice per group. n indicates the average number of nuclei counted per mouse.
(TIF)

S3 Fig Slowly proliferating progenitor/TA cells are not enriched in basal cell or luminal cell compartments of the prostate. Prostate tissue sections of 7 week old mice treated with CldU followed by prolonged treatment with IdU **(A)** were triple stained for CIdU, IdU and Krt14 and quantification of the labeled cells was performed in the Krt14-positive (basal) and the Krt14-negative (luminal) epithelial cell compartments. Here we show the graphic representation of the percentages of prostate cells labeled with CIdU, IdU, or CIdU/IdU in the basal or the luminal compartments of the distal/intermediate **(B)** and proximal **(C)** regions of prostatic ducts. The predicted stochastic fraction is also shown. Data represent the means *±* SD for three mice per group. n indicates the average number of nuclei counted per mouse.
(TIFF)

S4 Fig Treatment of the prostate epithelium with CldU and variable wash-out periods prior to IdU administration confirms that the renewal of the adult prostate epithelium does not depend on slowly serially proliferating progenitor/TA cells. Detection of CldU/IdU co-labeled cells was performed on different groups of 7 week old mice treated by sequential administration of CIdU and IdU interrupted with variable periods of wash-out as described in **(A)**. Mice were sacrificed immediately after the end of IdU treatment. **(B)** Tissue sections of the distal/intermediate regions of the prostate ducts were double stained for CIdU and IdU. Here we show the graphic representation of the percentages of prostate (basal and luminal) cells labeled with CIdU, IdU, or CIdU/IdU. Results are expressed as mean ± SD for three mice per group. n indicates the average number of nuclei counted per mouse.
(TIF)

S5 Fig Proliferating progenitor/TA cells are not enriched in the basal cell or luminal cell compartments in the regenerating prostate epithelium of castrated mice. Prostate tissue sections of the prostates of 7 week old castrated mice sequentially treated with CIdU and IdU (1 day each) at day 2 or day 3 after androgen supplementation **(A)** were triple stained for CIdU, IdU and Krt14 and quantification of the labeled cells was performed in the Krt14-positive (basal) and the Krt14-negative (luminal) epithelial cell compartments. **(B, C)** Here we show the graphic representation of the percentages of prostate cells labeled with CIdU, IdU, or CIdU/IdU in the basal or the luminal compartments of the distal/intermediate **(B)** and proximal **(C)** regions of prostatic ducts. 2dR and 3dR indicate mice that were treated with the thymidine analogs at day 2 or day 3 after androgen supplementation, respectively. The predicted stochastic fraction is also shown. Data represent the mean *±* SD for three mice per group. n indicates the average number of nuclei counted per mouse.
(TIF)
