# Supplementary figures and images for: Cell Kinetic Studies Fail to Identify Sequentially Proliferating Progenitors as the Major Source of Epithelial Renewal in the Adult Murine Prostate
Source: PLoS One. 2015 May 29;10(5):e0128489. doi: 10.1371/journal.pone.0128489 (PMC4449166; doi:10.1371/journal.pone.0128489)

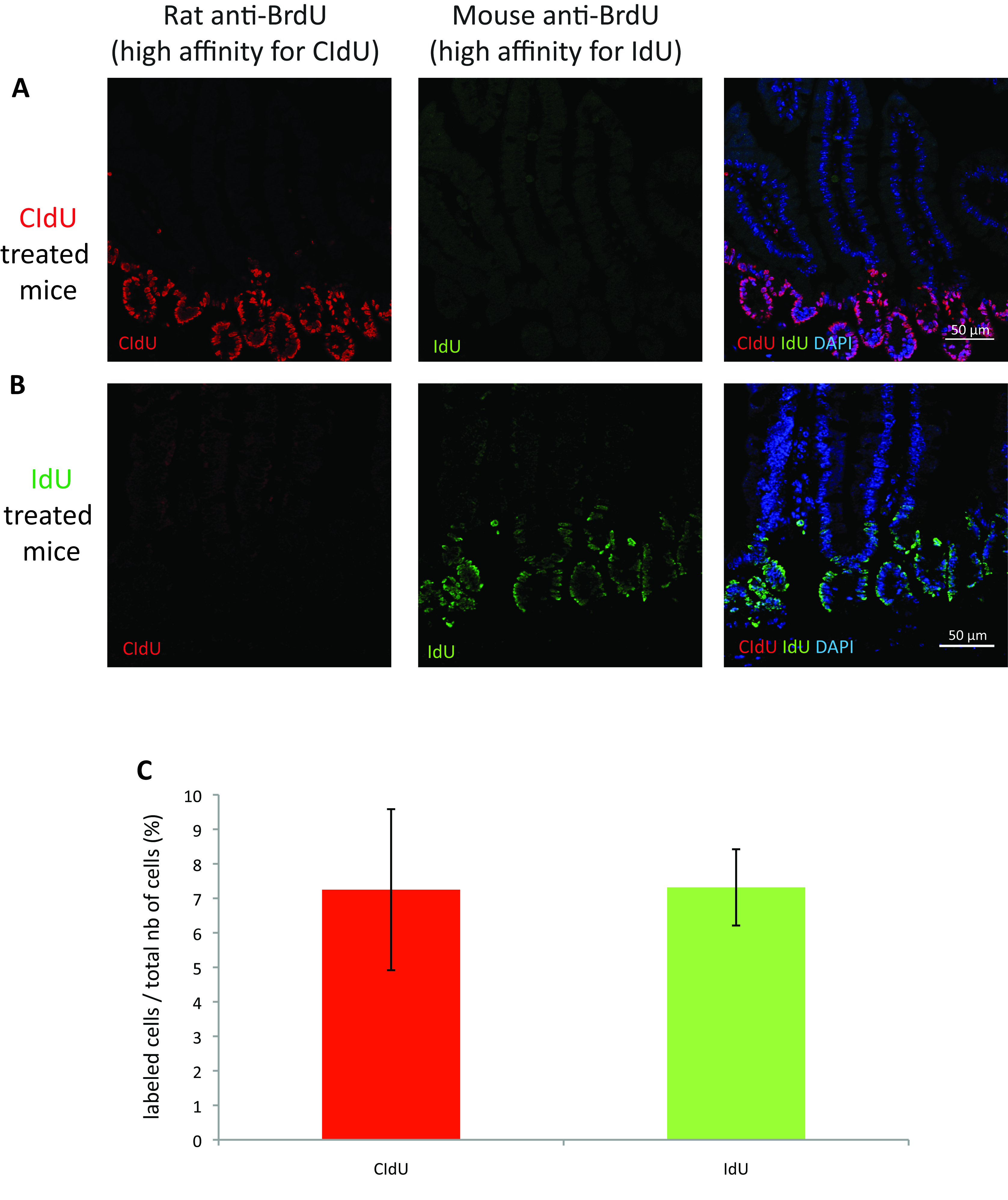

Supplement: S1 Fig — To this end, 7 week old male mice were treated for 1 day with CldU (A) or IdU (B) and small intestine tissue sections were double immunostained with the rat anti-BrdU antibody (high affinity for CIdU) and the mouse anti-BrdU antibody (high affinity for IdU) as described in the materials and methods section. The mouse anti-BrdU antibody produces no signal in mice treated with CldU (A, middle panel) and the rat anti-BrdU antibody produces no signal in mice treated with IdU (B, left panel). (C): Incorporation of the thymidine analogs, CldU and IdU occurs at a similar rate in the mouse prostate. Graphic representation of the percentages of epithelial prostate cells (both basal and luminal) labeled with either CIdU or IdU in 7 week old mice treated for 1 day with CldU or IdU. Data represent the mean ± SD for three mice per group. (TIF) [file pone.0128489.s001.tif]

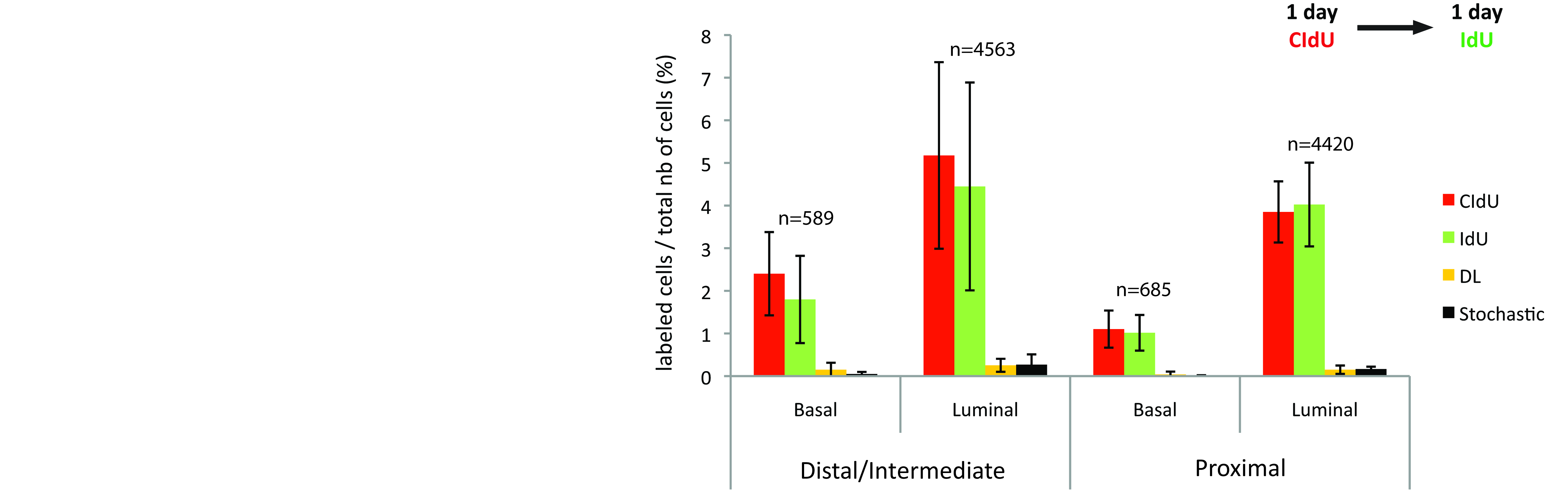

Supplement: S2 Fig — Prostate tissue sections of 7 week old mice sequentially treated with CIdU and IdU for 1 day each were triple stained for CIdU, IdU and Krt14 and quantification of the labeled cells was performed in the Krt14-positive (basal) and the Krt14-negative (luminal) epithelial cell compartments. Here we show the graphic representation of the percentages of prostate cells labeled with CIdU, IdU, or CIdU/IdU in the basal or the luminal compartments of the distal/intermediate and proximal regions of prostatic ducts. The predicted stochastic fraction is also shown. Data represent the means ± SD for three mice per group. n indicates the average number of nuclei counted per mouse. (TIF) [file pone.0128489.s002.tif]

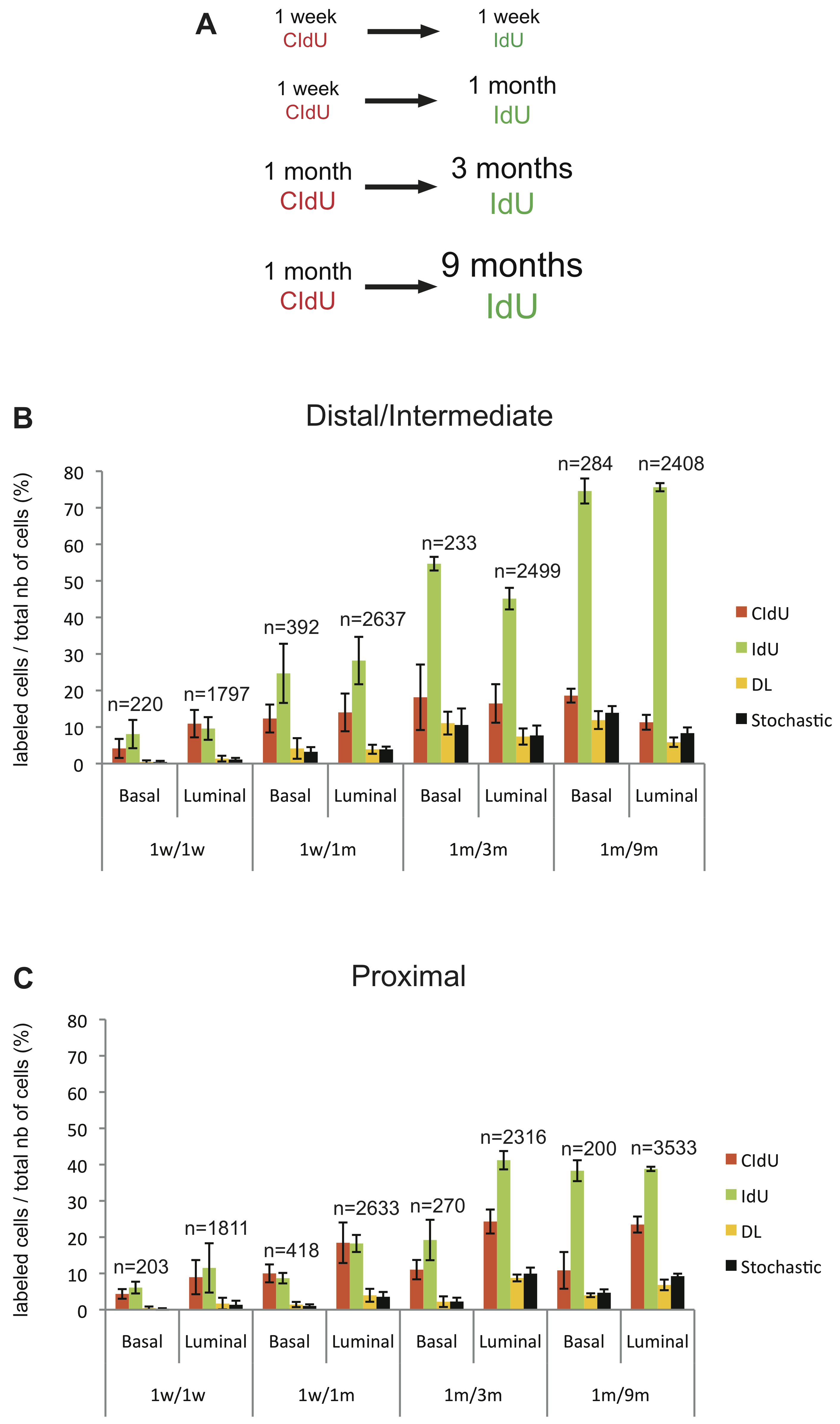

Supplement: S3 Fig — Prostate tissue sections of 7 week old mice treated with CldU followed by prolonged treatment with IdU (A) were triple stained for CIdU, IdU and Krt14 and quantification of the labeled cells was performed in the Krt14-positive (basal) and the Krt14-negative (luminal) epithelial cell compartments. Here we show the graphic representation of the percentages of prostate cells labeled with CIdU, IdU, or CIdU/IdU in the basal or the luminal compartments of the distal/intermediate (B) and proximal (C) regions of prostatic ducts. The predicted stochastic fraction is also shown. Data represent the means ± SD for three mice per group. n indicates the average number of nuclei counted per mouse. (TIFF) [file pone.0128489.s003.tiff]

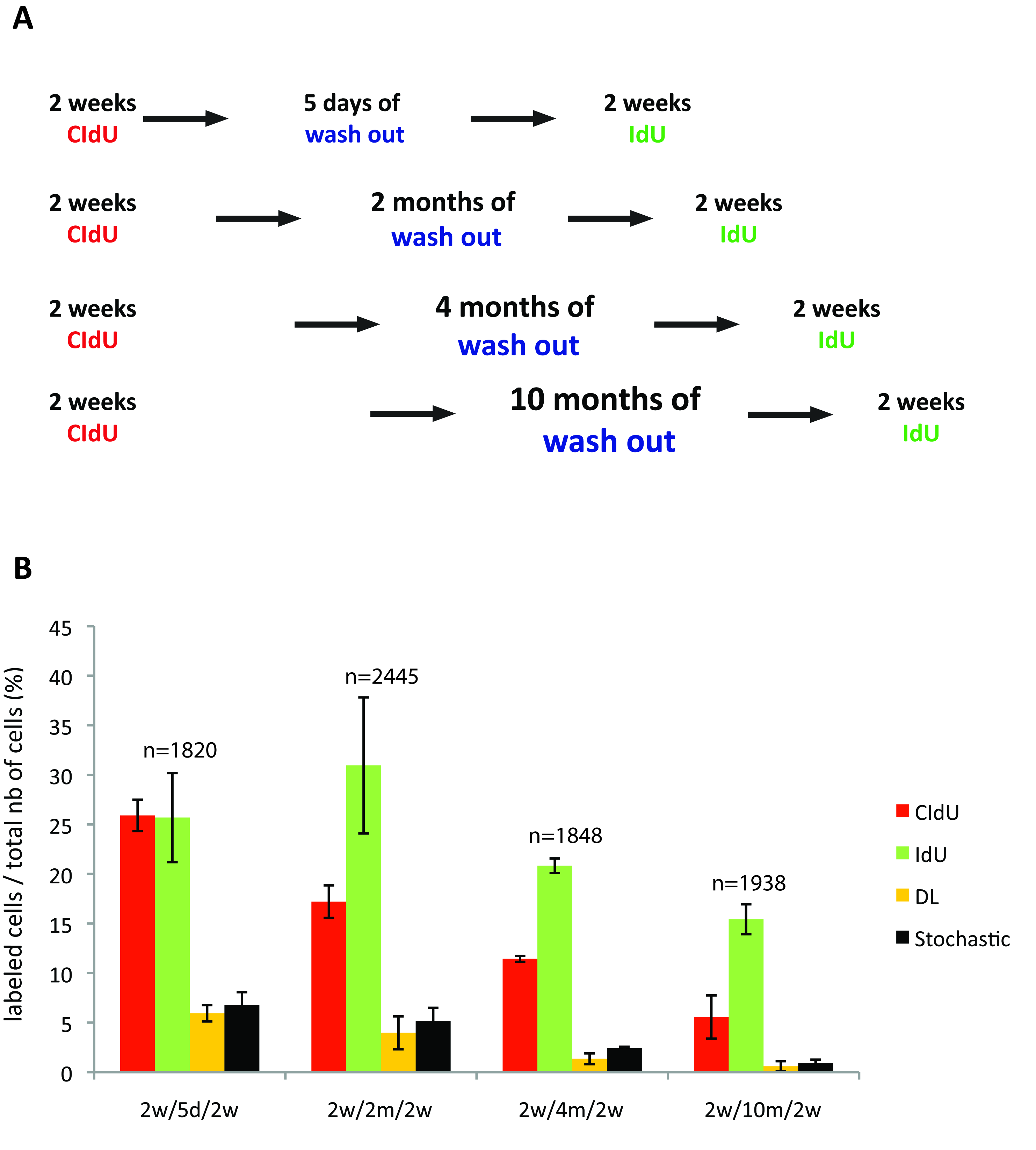

Supplement: S4 Fig — Detection of CldU/IdU co-labeled cells was performed on different groups of 7 week old mice treated by sequential administration of CIdU and IdU interrupted with variable periods of wash-out as described in (A). Mice were sacrificed immediately after the end of IdU treatment. (B) Tissue sections of the distal/intermediate regions of the prostate ducts were double stained for CIdU and IdU. Here we show the graphic representation of the percentages of prostate (basal and luminal) cells labeled with CIdU, IdU, or CIdU/IdU. Results are expressed as mean ± SD for three mice per group. n indicates the average number of nuclei counted per mouse. (TIF) [file pone.0128489.s004.tif]

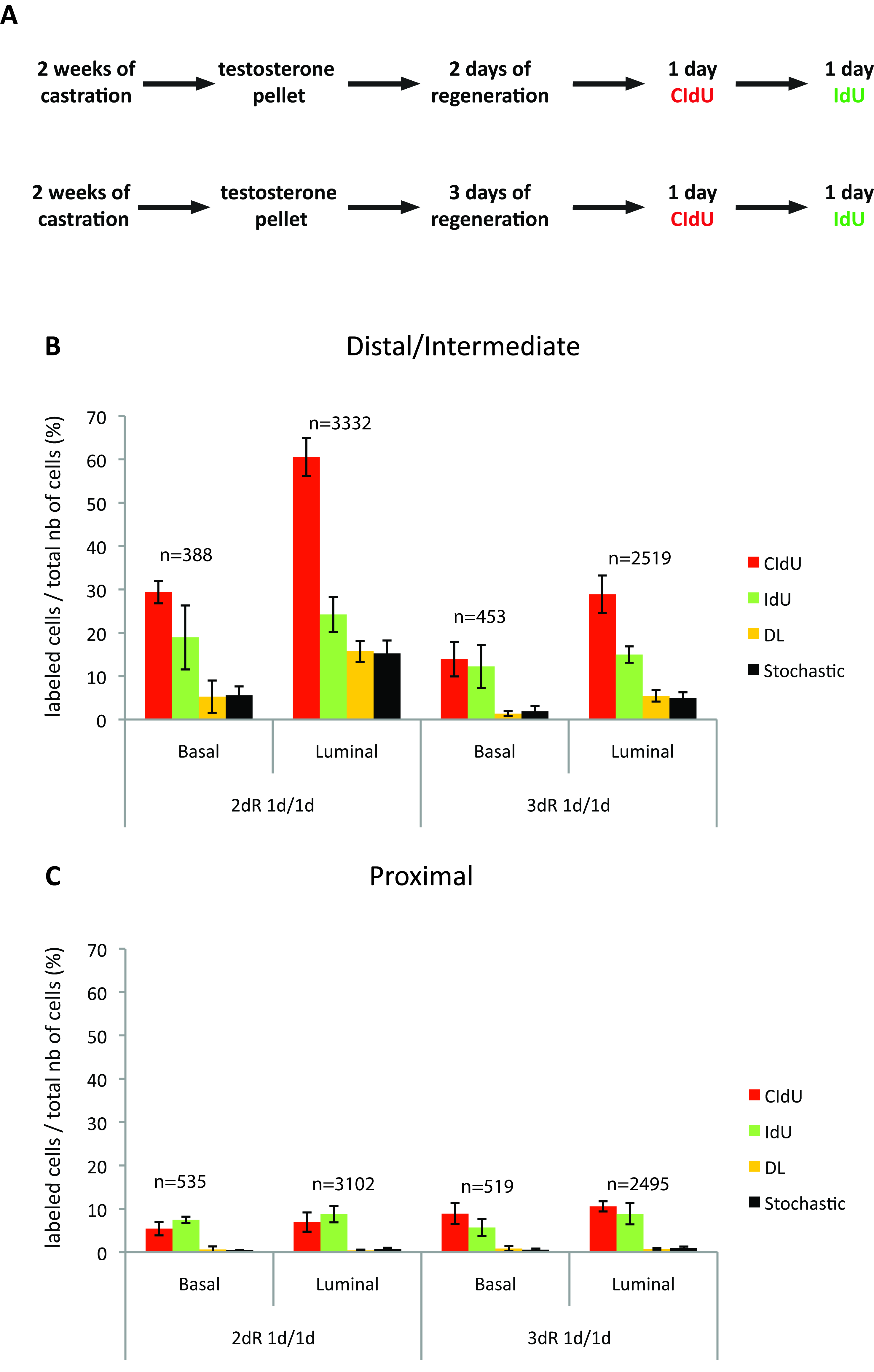

Supplement: S5 Fig — Prostate tissue sections of the prostates of 7 week old castrated mice sequentially treated with CIdU and IdU (1 day each) at day 2 or day 3 after androgen supplementation (A) were triple stained for CIdU, IdU and Krt14 and quantification of the labeled cells was performed in the Krt14-positive (basal) and the Krt14-negative (luminal) epithelial cell compartments. (B, C) Here we show the graphic representation of the percentages of prostate cells labeled with CIdU, IdU, or CIdU/IdU in the basal or the luminal compartments of the distal/intermediate (B) and proximal (C) regions of prostatic ducts. 2dR and 3dR indicate mice that were treated with the thymidine analogs at day 2 or day 3 after androgen supplementation, respectively. The predicted stochastic fraction is also shown. Data represent the mean ± SD for three mice per group. n indicates the average number of nuclei counted per mouse. (TIF) [file pone.0128489.s005.tif]
